# Correction: Yagolovich et al. DR5-Selective TRAIL Variant DR5-B Functionalized with Tumor-Penetrating iRGD Peptide for Enhanced Antitumor Activity against Glioblastoma. *Int. J. Mol. Sci.* 2022, *23*, 12687

**DOI:** 10.3390/ijms25105334

**Published:** 2024-05-14

**Authors:** Anne V. Yagolovich, Alina A. Isakova, Artem A. Artykov, Yekaterina V. Vorontsova, Diana V. Mazur, Nadezhda V. Antipova, Marat S. Pavlyukov, Mikhail I. Shakhparonov, Anastasia M. Gileva, Elena A. Markvicheva, Ekaterina A. Plotnikova, Andrey A. Pankratov, Mikhail P. Kirpichnikov, Marine E. Gasparian, Dmitry A. Dolgikh

**Affiliations:** 1 Shemyakin-Ovchinnikov Institute of Bioorganic Chemistry RAS, 117997 Moscow, Russia; 2 Faculty of Biology, Lomonosov Moscow State University, 119192 Moscow, Russia; 3 Manebio LLC, 115280 Moscow, Russia; 4 National Medical Research Radiological Centre of the Ministry of Health of the Russian Federation, P.A. Hertsen Moscow Oncology Research Institute, 125284 Moscow, Russia

In the original publication [1], there was a mistake in Figure 4D as published. Due to the similar lack of cytotoxic effects, the control 0.000 nM/DR5-B image was accidentally replaced with a part of the 0.005 nM/DR5-B image. However, the error does not have a critical impact on the results, since the lack of cytotoxicity of 0.005 nM DR5-B is also confirmed by the viability data (see Figure 4B). The authors sincerely apologize for the confusion. The corrected Figure 4 appears below.

To specifically clarify the conflicts of interest in the original publication [1], the corrected Conflicts of Interest appears here. The authors state that the scientific conclusions are unaffected. This correction was approved by the Academic Editor. The original publication has also been updated.

## Figures and Tables

**Figure 4:**
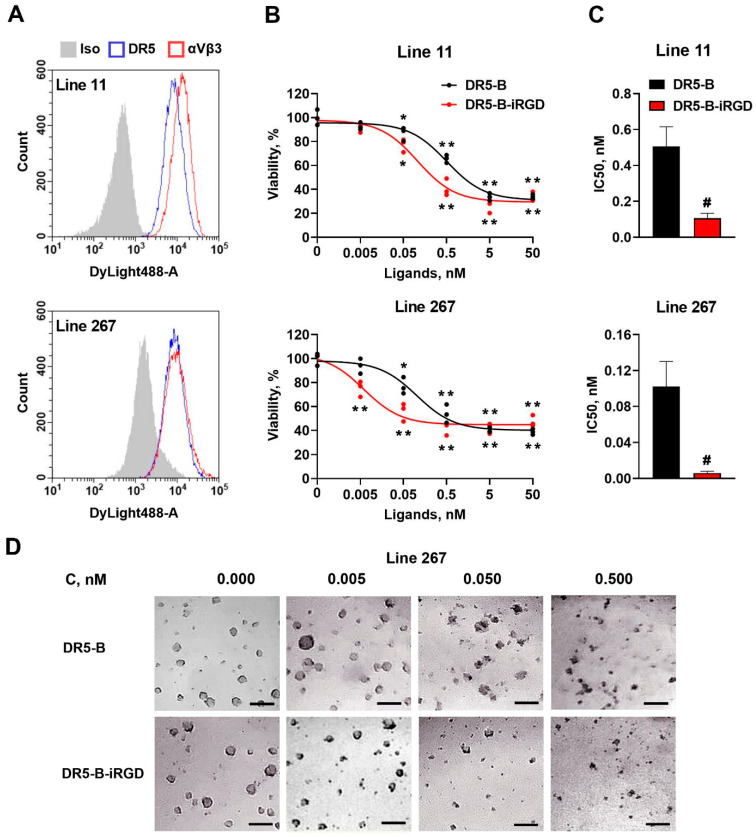
Cytotoxicity of DR5-B and DR5-B–iRGD in primary human glioblastoma patient-derived neurospheres. (**A**) Surface expression of the DR5 and integrin αvβ3 receptors determined by flow cytometry. (**B**) Primary human glioblastoma cell lines 11 and 267 were incubated with DR5-B or DR5-B–iRGD for 72 h, and viability was analyzed by the Alamar Blue assay. The data were displayed as mean ± SD from at least three replicates. * *p* < 0.005 and ** *p* < 0.0005 indicate significant difference from the control according to one-way ANOVA followed by Dunnett’s post hoc test. (**C**) IC50 values of DR5-B and DR5-B–iRGD were determined as the drug concentrations resulting in the 50% inhibition of cell growth by nonlinear regression in GraphPad Prism 8 software. # *p* < 0.005 indicates significant difference from the control according to Student’s *t*-test. (**D**) Morphology of primary patient-derived neurospheres (Line 267) treated with DR5-B or DR5-B-iRGD. Scale bar is 200 µm.
